# Hormonal status affects plasma exposure of tamoxifen and its main metabolites in tamoxifen-treated breast cancer patients

**DOI:** 10.1186/s40360-019-0358-y

**Published:** 2019-12-19

**Authors:** João Paulo Bianchi Ximenez, Jurandyr Moreira de Andrade, Maria Paula Marques, Eduardo Barbosa Coelho, Guilherme Suarez-Kurtz, Vera Lucia Lanchote

**Affiliations:** 1 Department of Clinical Analysis, Toxicology and Food Science, School of Pharmaceutical Sciences of Ribeirão Preto, University of São Paulo, Ribeirão Preto, Brazil; 2 Department of Gynecology and Obstetrics, Ribeirão Preto Medical School, University of São Paulo, Ribeirão Preto, Brazil; 3 Department of Internal Medicine, Ribeirão Preto Medical School, University of São Paulo, Ribeirão Preto, Brazil; 4 Division of Pharmacology, National Cancer Institute of Brazil, Rio de Janeiro, Brazil

**Keywords:** Tamoxifen, Endoxifen, Hormonal status, CYP2D6, CYP3A4/5, Breast Cancer

## Abstract

**Background:**

Tamoxifen is considered a prodrug of its active metabolite endoxifen, which is dependent on the CYP2D6 and CYP3A enzymes. Tamoxifen pharmacokinetic variability influences endoxifen exposure and, consequently, its clinical outcome. This study investigated the impact of hormonal status on the pharmacokinetics of tamoxifen and its metabolites in TAM-treated breast cancer patients.

**Methods:**

TAM-treated breast cancer patients (*n* = 40) previously believed to have CYP3A activity within the normal range based on oral midazolam and phenotyped as CYP2D6 normal metabolizers using oral metoprolol were divided into two groups according to premenopausal (*n* = 20; aged 35–50 years) or postmenopausal (n = 20; aged 60–79 years) status. All patients were treated with 20 mg/day tamoxifen for at least three months. Serial plasma samples were collected within the 24 h dose interval for analysis of unchanged tamoxifen, endoxifen, 4-hydroxytamoxifen and N-desmethyltamoxifen quantified by LC-MS/MS. CYP activities were assessed using midazolam apparent clearance (CYP3A) and the metoprolol/alfa-hydroxymetoprolol plasma metabolic ratio (CYP2D6). CYP3A4, CYP3A5 and CYP2D6 SNPs and copy number variation were investigated using TaqMan assays.

**Results:**

Postmenopausal status increased steady-state plasma concentrations (Css) of tamoxifen (116.95 vs 201.23 ng/mL), endoxifen (8.01 vs 18.87 ng/mL), N-desmethyltamoxifen (485.16 vs 843.88 ng/mL) and 4-hydroxytamoxifen (2.67 vs 4.11 ng/mL). The final regression models included hormonal status as the only predictor for Css of tamoxifen [β-coef ± SE, *p*-value (75.03 ± 17.71, *p* = 0.0001)] and 4-hydroxytamoxifen (1.7822 ± 0.4385, *p* = 0.0002), while endoxifen Css included hormonal status (8.578 ± 3.402, *p* = 0.02) and race (11.945 ± 2.836, *p* = 0.007). For N-desmethyltamoxifen Css, the final model was correlated with hormonal status (286.259 ± 76.766, *p* = 0.0007) and weight (− 8.585 ± 3.060, *p* = 0.008).

**Conclusion:**

The premenopausal status was associated with decreased endoxifen plasma concentrations by 135% compared to postmenopausal status. Thus, the endoxifen plasma concentrations should be monitored mainly in the premenopausal period to maintain plasma levels above the efficacy threshold value.

**Trial registration:**

RBR-7tqc7k.

## Background

Tamoxifen (TAM) has been used for more than 40 years to treat early breast cancer and metastatic breast cancer in either preoperative or postoperative adjuvant therapy. A selective ER modulator, TAM is one of the most commonly used endocrine therapeutic agents for the treatment of estrogen receptor (ER)-positive breast carcinoma, acting as an estrogen antagonist or agonist depending on tissue type [1]. In adjuvant treatment, 5-year TAM therapy almost halves the annual risk of breast cancer recurrence and decreases the breast cancer mortality incidence by one-third in pre- and postmenopausal patients [2].

The TAM anticancer effect is due to its two active metabolites, 4-hydroxytamoxifen (4-HTAM) and endoxifen (END), which have a 100-fold higher antiestrogenic effect than the parent drug [3]. Once END plasma concentrations exceed 4-HTAM concentrations, END is considered the metabolite responsible for the clinical impact of TAM. Therefore, there is considerable concern regarding END exposure variability and consequently treatment outcome [4].

TAM is mainly converted by CYP3A4/5 to N-desmethyltamoxifen (NDTAM), and it is subsequently converted to END by CYP2D6. As a minority pathway, TAM is oxidized by CYP2C9 and CYP2D6 to 4-HTAM, which is further converted to END by CYP3A4/5 [5, 6]. TAM and its oxidized metabolites are further metabolized by phase II enzymes, such as sulfotransferases (SULTs) and UDP-glucuronosyltransferases UGTs [7].

The potential role of CYP2D6 genotype assessment in determining whether breast cancer patients should receive TAM is controversial. Multiple studies [8–11] have shown that the clinical outcome of adjuvant TAM is influenced by the CYP2D6 genotype since poor metabolizers (PMs) have a higher risk of breast cancer recurrence than normal metabolizers (NMs). However, other clinical trials, including the ATAC [12], BIG1–98 [13] and ABCSG8 [14] trials, have shown conflicting data from 5-year TAM prospective analysis regarding the association between CYP2D6 genotype and clinical outcome.

Based on current evidence [1], CYP2D6 NMs and ultrarapid metabolizers (UMs) are expected to achieve therapeutic END plasma concentrations following administration of TAM at 20 mg/day standard doses. For CYP2D6 intermediate metabolizers (IMs) and PMs, 40 mg/day TAM can be considered or alternative therapy, such as an aromatase inhibitor (AI) for postmenopausal women or AI along with ovarian function suppression in premenopausal women [1].

Regarding the role of measurement of END concentrations, lower END concentrations were shown to be associated with poor clinical outcome in a mixed cohort of pre- and postmenopausal [4] patients and in a study of only premenopausal patients [15]. Notably, an in vitro study showed that END could block breast cancer cell growth in the presence of a high estrogen concentration that mimics premenopausal patients [16].

In addition to the pharmacogenetic effect, there has been speculation regarding the association between menopausal status and TAM treatment outcome. Since the metabolism of drugs is associated with hormonal status [17–20], plasma concentrations of TAM active metabolites could be influenced by the menopausal status of the women.

In postmenopausal women, TAM and its metabolites occupy most of the available ERs, suggesting that variation in END plasma concentration would have a small effect in blocking these receptors [21]. However, END may be critical to saturate the ERs in premenopausal women, in whom TAM and its metabolites are estimated to occupy only 90–95% of the available receptors [21].

The current scenario points to the need for combined pharmacokinetic and pharmacogenetic analyses in both pre- and postmenopausal patients since many of the studies have reported results from only one hormonal status group. This study investigates the influence of menopausal status on the pharmacokinetics of TAM and its metabolites END, 4-HTAM and NDTAM in TAM-treated breast cancer patients phenotyped as NMs for CYP2D6 and with CYP3A activity based on the oral midazolam clearance.

## Methods

### Patients and data collection

A total of 40 TAM-treated breast cancer patients (20 mg/day) were recruited, and they were distributed into 2 groups according to age and menopausal status (*n* = 20/group): A) premenopausal (patients aged < 50 years) and B) postmenopausal (patients aged > 60 years) groups. All patients were histologically diagnosed with ER-positive breast cancer, and the clinical diagnosis of menopause was based on menstrual history, ultrasound features (ovarian volume) and age. Patients were suitable for inclusion if they were not concomitantly taking moderate or potent inhibitors or inducers of CYP3A and the drug transporter ABCB1 (P-glycoprotein) or inhibitors of CYP2D6. The study was approved by the ethics review committees of the School of Pharmaceutical Sciences of Ribeirão Preto, University of São Paulo, SP, Brazil and of the Teaching Hospital of Ribeirão Preto Medical School, University of São Paulo, SP, Brazil (record number: 35539714.7.0000.5403). All patients provided written consent.

TAM pharmacokinetics and metabolism were evaluated after at least three months of drug treatment, at steady-state, during a 24 h dosing interval. Blood samples (1 mL) were collected predosing and 30 min and 1, 1.5, 2, 3, 4, 6, 8, 12, 16 and 24 h after the administration of the daily TAM dose. Twenty-four hours after the end of the TAM study, metoprolol (100 mg) and midazolam (15 mg) were orally administered as a single dose, and blood samples (4 mL) were collected predosing and 15 and 30 min and 1, 2, 3, 4, 5 and 6 h after drug administration. For pharmacogenetic screening, whole blood (2 mL) was collected in EDTA tubes and stored at − 70 °C until analysis.

### TAM and its metabolites measurement in plasma

Plasma samples of 200 μL were added with the internal standard solution (mexiletine, 5 μg/mL), 25 μL of 1 M sodium hydroxide aqueous solution and 2 mL methyl tert-butyl ether. TAM, NDTAM, 4-HTAM and END were resolved on an RP-Select B LiChroCART® C18 column using as a mobile phase a mixture of 10 mM ammonium formate and acetonitrile (1:1, v:v) added with 0.1% formic acid. TAM and its metabolites were quantified by Quattro Micro LC triple-quadrupole mass spectrometry (Waters, Milford, EUA) in the positive ion electrospray ionization mode as described previously with some modifications [22]. The method had no matrix effect and showed linearities for TAM and END in the range of 1–1250 ng/mL, for 4-HTAM of 0.4–500 ng/mL and for NDTAM of 2–2500 ng/mL of plasma. The coefficients of variation and the relative standard errors of the accuracy and precision studies were less than 15%.

### CYP2D6 phenotype

Aliquots of 100 μL of plasma supplemented with tramadol as an internal standard (50 μg/ml), 25 μL of 1 M sodium hydroxide aqueous solution, 10 mg of sodium chloride and 2 mL of dichloromethane-diisopropyl ether (1:1, v/v). The compounds were separated on an RP Select B column (LiChrospher®60 Merck, Darmstadt, Germany) using buffer phosphate (0.05 M and pH 3.5) and acetonitrile (90:10, v/v) as the mobile phase and analyzed using a fluorescence detector (229 and 298 nm) [23, 24]. Calibration curves were constructed from 20 to 1000 ng metoprolol/mL and from 10 to 500 ng α-hydroxymetoprolol/mL. Accuracy and precision studies showed coefficients of variation and relative standard errors less than 15%.

### CYP3A4 in vivo activity

Aliquots of 1 mL plasma supplemented with clobazam (2.5 ng) as an internal standard were extracted with toluene-isoamyl alcohol (100:0.1, v/v). The compounds were separated on a Purospher RP 18e column (MerckKGaA, Darmstadt, Germany) using acetonitrile:10 mmol/L ammonium acetate aqueous solution (1:1, v/v) as the mobile phase and quantified by Quattro Micro LC triple-quadrupole mass spectrometry (Waters, Milford, USA) in the positive ion electrospray ionization mode [25]. Calibration curves were constructed from 0.1 to 50 ng/mL plasma. The method showed coefficients of variation and relative standard errors less than 15%, respectively, in the studies of precision and accuracy.

### Genotyping

DNA was obtained from peripheral blood leukocytes according to usual procedures with a QIAamp DNA Blood Mini Kit (Qiagen, Hilden, GER) according to the manufacturer’s instructions.

The following SNPs were detected by real-time polymerase chain reaction with 5-nuclease allelic discrimination assays according to the manufacturer’s instructions: CYP2D6 -1584C > G (rs1080985), 31G > A (rs769258), 100C > T (rs1065852), 1023C > T (rs28371706), 1846G > A (rs3892097), 2549A > del (rs35742686), 2615_2617delAAG (rs28371720), 2850C > T (rs16947), 2988G > A (rs28371725), 3183G > A (rs59421388), 4180G > C (rs1135840), CYP3A4*1B (rs2740574), CYP3A4*22 (rs35599367), and CYP3A5*3 (rs776746). Gene deletion (CYP2D6*5, Hs00010001_cn) and duplication/multiplication (CYP2D6*xN) were analyzed by TaqMan copy number assay.

### Pharmacokinetic analyses

Pharmacokinetic analyses of TAM and its metabolites were performed as a non-compartmental model using WinNonlin 4.4 (Pharsight Corporation, Mountain View, CA, USA). The observed maximum plasma concentration (Cmax) and the time to reach the Cmax (Tmax) were obtained by visual inspection of the experimental data. The area under the plasma concentration-time curve from time zero to 24 h (AUCτ) was calculated using linear trapezoidal rule. The concentration in the steady state (Css) was calculated by dividing the AUCτ by τ, where τ is the dosing interval. The metabolic ratio (MR) was defined as the ratio of AUCτ parent drug to AUCτ metabolite drug.

The metoprolol oxidation capacity was expressed according to the log10 metoprolol/α-hydroxymetoprolol plasma ratio (metabolic ratio - MR). Poor metabolizers (PM) were identified in situations where log10 metoprolol/α-hydroxymetoprolol plasma ratios were ≥ 1.5 [23]

CYP3A activity was estimated by the apparent clearance (CL/F) of midazolam evaluated by the software WinNonlin 4.4., corrected for body weight and expressed as mL min^− 1^ kg^− 1^, according to the Lamba et al. (2002) [26].

### Statistical analyses

Based on a previous study reported by Etienne et al., 1989 [27], twenty patients in each group (pre- and postmenopausal) were estimated to be adequate to detect a 30% change in the AUC of TAM between the two study groups, with a power of at least 80% and alpha level of 5%.

Age (years), weight, BMI, log of MR (metoprolol/α-hydroxymetoprolol), apparent midazolam clearance, AUCτ, C_SS_ and MR of TAM and its metabolites were analyzed as continuous variables; hormonal status (pre- and postmenopausal), race (white or non-white), CYP2D6 (NM, IM, and others), CYP3A5*3 (expressors and non-expressors), CYP3A4*22 (carriers and non-carriers) and CYP3A4*1B (carriers and non-carriers) were analyzed as categorical variables.

CYP2D6 diplotypes were inferred using HaploStats software (version 1.7.7) implemented on the R platform. Software-generated haplotypes were compared to the CYP Allele Nomenclature Database for the star (*) allele designation. Haplotypes not matched with known CYP2D6 alleles were grouped into the “other” category [28]. The activity score (AS) system was used to define the perceived functionality of the CYP2D6 diplotypes. Values of 0–2 were attached to the alleles identified in the study cohort as follows: zero, no-function alleles (*4, *4xN, *5); 0.5, decreased-function alleles (*9, *10, *17, *29, *41); 1, normal-function alleles (*1, *2, *39) and 2, increased-function alleles (*1xN, *2xN). The AS of diplotypes resulted from the sum of the assigned value to each allele. Patients with AS = 0, AS = 0.5, and AS > 2 were designated genetic PM, IM, and UM, respectively. Patients with AS = 1, AS = 1.5, and AS = 2 were designated NM [29, 30].

First, descriptive statistics were run to describe the general characteristics of the participants. Then, AUCτ, C_SS_ and MR of TAM and its metabolites were compared among the categorical variables by t-test. Subsequently, Spearman correlation rank tests were used to examine the correlations among TAM and its metabolites AUCτ, C_SS_ and MR with genotype, phenotype and demographic variables. Finally, multiple regression modeling was used to assess the effect of genotypes, phenotypes and demographic variables on the Css of TAM and its metabolites. The genotype frequencies of the *CYP2D6*, CYP3A5*3, CYP3A4*22 and CYP3A4*1B polymorphisms were derived by gene counting. Deviations from Hardy–Weinberg equilibrium were assessed by the chi-square test. The statistical analyses were performed with RStudio version 1.1.456 (RStudio, Boston, MA), and all tests considered *p* <  0.05 statistically significant.

## Results

### Study data

The data for pharmacokinetics analysis consisted of 480 samples collected from 40 TAM-treated breast cancer patients distributed into two groups according to age and menopausal status. The characteristics of the patients are shown in Table 1. The cohort study showed a geometric mean age of 45.3 years and a body mass index (BMI) of 29.58 for the premenopausal group (*n* = 20). For the postmenopausal group (n = 20), the geometric mean of age was 66.8 years, and BMI was 28.07.

**Table 1:** Baseline demographics and phenotypes in the investigated TAM-treated breast cancer patients (*n* = 40). Data are reported as the mean and 95% CI or percent

	Pre-menopausal (*n* = 20)	Post-menopausal (*n* = 20)
Age at diagnosis (years)	45.3 (44.70–45.90)	66.8 (66.13–67.46)
BMI (kg/m^2^)	29.58 (28.90–30.25)	28.07 (27.58–28.56)
Color (%)	white (85), non-white (15)	white (75), non-white (25)
Cancer type (%)	ductal in situ (5), invasive ductal (90) and invasive lobular (5)	ductal in situ (0), invasive ductal (90) and invasive lobular (10)
HER2 status (%)	positive (20), negative (75) and unknown (5)	positive (10), negative (80) and unknown (10)
Chemotherapy (%)	Yes (20) and No (80)	Yes (5) and No (95)
CYP2D6 phenotype (MR)	0.25 (0.21–0.30)	0.24 (0.19–0.29)
Midazolam oral clearance (mL/min/kg)	30.09 (21.55–42.02)	24.10 (19.41–29.92)

*MR* Metabolic ratio (plasma log metoprolol/α-hydroxymetoprolol)

### Influence of hormonal status on the pharmacokinetics of tamoxifen and its metabolites

**Fig. 1:**
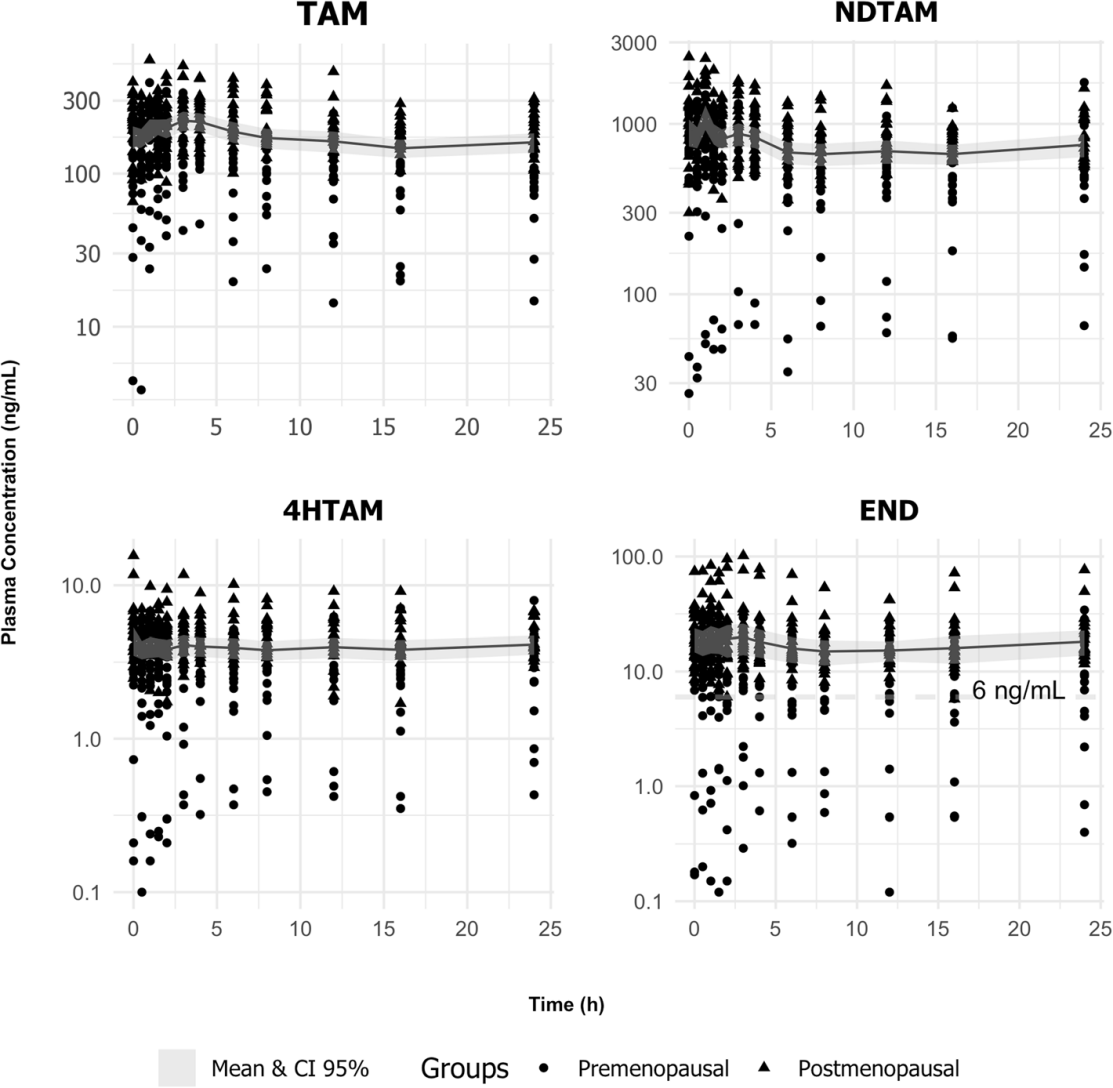
Plasma concentration versus time of TAM and NDTAM (upper panels) and 4-HTAM and END (lower panels) observed during a dosing interval of TAM therapy (20 mg/day) in all investigated TAM-treated breast cancer patients (*n* = 40). The value of 6 ng/ml END corresponds to the threshold associated with efficacy

The mean plasma concentration–time profiles for TAM and its metabolites END, 4-HTAM and NDTAM in all investigated (*n* = 40) TAM-treated breast cancer patients given 20 mg daily are shown in Fig. 1. The fluctuation values at steady state, calculated as the ratio (C_max_-C_min_)/C_min_ and expressed as the geometric mean and 95% CI, were 206% (176–235) for TAM, 156% (148–164) for NDTAM, 127% (116–138) for 4-HTAM and 154% (139–169) for END.

**Fig. 2:**
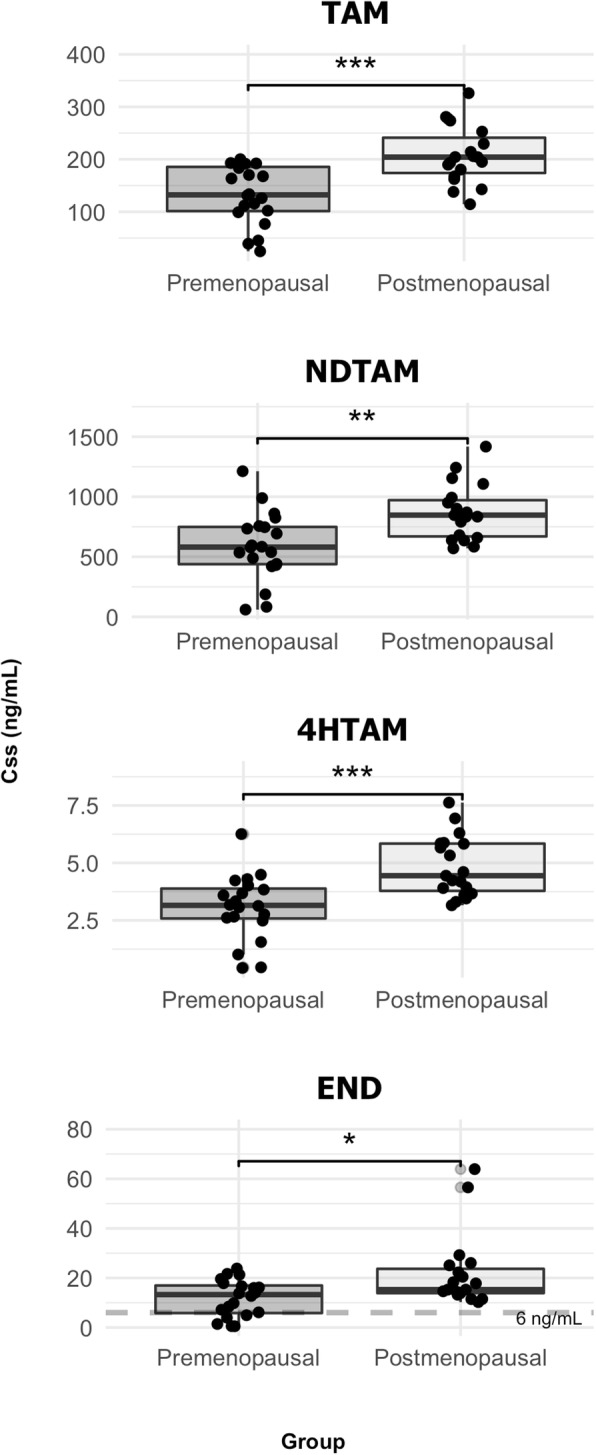
Whisker box-plots depicting the hormonal status influence on TAM and NDTAM (upper panel) and 4-HTAM and END (lower panel) concentrations at steady-state. The value of 6 ng/ml END corresponds to the threshold associated with efficacy. * *p* < 0.05; ** *p* < 0.01; *** *p* < 0.001

The pharmacokinetic parameters of TAM and its metabolites, evaluated at steady state, are summarized in Table 2 and Fig. 2 according to hormonal status, which was defined as premenopausal (*n* = 20) or postmenopausal (n = 20). The metabolic ratios (MR) did not differ between groups (*p* > 0.05), with values of TAM to NDTAM of approximately 0.20, while NDTAM to END was higher than 40. However, the hormonal status increased the steady-state plasma concentration (C_SS_) of TAM and its metabolites NDTAM and 4-HTAM by 70–80%, whereas END was increased by 135% (Table 2).

**Table 2:** Effect of hormonal status on the TAM pharmacokinetics and its metabolites END, 4-HTAM and NDTAM in the investigated TAM-treated breast cancer patients (*n* = 40). Data are reported as the geometric mean (95% CI)

	Premenopausal (n = 20)			Postmenopausal (n = 20)			
AUCτ (ng*h/mL)	Geometric Mean	95% CI		Geometric Mean	95% CI		*P*-value
TAM	2806.78	2128.19	3701.75	4829.41	4244.20	5495.30	< 0.001
NDTAM	11,643.86	8132.88	16,670.55	20,253.09	17,886.43	22,932.88	< 0.010
4-HTAM	61.67	44.15	86.15	112.23	98.75	127.55	< 0.001
END	192.14	111.75	330.35	452.77	355.65	576.40	0.013
Css (ng/mL)							
TAM	116.95	88.67	154.24	201.23	176.84	228.97	< 0.001
NDTAM	485.16	338.87	694.61	843.88	745.27	955.54	< 0.010
4-HTAM	2.57	1.84	3.59	4.68	4.11	5.31	< 0.001
END	8.01	4.66	13.76	18.87	14.82	24.02	0.013
Metabolic Ratios							
TAM/NDTAM	0.20	0.15	0.26	0.21	0.19	0.23	0.523
TAM/4-HTAM	46.27	36.67	58.39	38.12	31.79	45.70	0.177
NDTAM/END	68.98	46.26	102.86	44.35	32.78	60.01	0.230
4-HTAM/END	0.30	0.20	0.44	0.24	0.19	0.31	0.275
Apparent clearance (L/h)							
TAM	7.13	5.40	9.40	4.14	3.64	4.71	0.015

Tamoxifen (TAM), endoxifen (END), 4-hydroxytamoxifen (4-HTAM) and N-desmethyl tamoxifen (NDTAM)

### Effect of CYP2D6 and CYP3A phenotypes on the disposition of tamoxifen and its metabolites

All 40 investigated TAM-treated breast cancer patients were classified as normal metabolizers (NM) for CYP2D6 activity based on the ratio log metoprolol/hydroxymetoprolol concentrations in plasma at 3 h > 1.5 (23). The log metoprolol/hydroxymetoprolol ratios in plasma (0.25 versus 0.24; Table 1) did not differ significantly across the two study groups (t-test, *p* = 0.7803).

The oral midazolam clearance varied 22-fold in all investigated patients (8.26–180.82 mL/min/kg). The oral midazolam clearance values were within the 10–40 mL/min/kg range for 29 patients (72%), below 10 mL/min/kg for one patient and higher than 40 mL/min/kg for ten patients. The t-test revealed no significant differences in the midazolam CL/F (30.09 versus 24.10 mL/min/kg; Table 1) across the two study groups (t-test, *p* = 0.1499).

### Association of *CYP2D6*, *CYP3A4* and *CYP3A5* polymorphisms on the plasma concentrations of TAM and its metabolites

The activity scores of the allelic frequencies of CYP3A4, CYP3A5 and CYP2D6 are shown in Table 3. No patients had a PM or UM CYP2D6 phenotype. All investigated patients were CYP3A4*1 carriers. Twenty-seven patients were CY3A5 non-expressors (*3/*3), while thirteen were genotyped as CY3A5 expressors (*1/*1 and *1/*3). Significant correlations between the polymorphic variants in CYP3A4, CYP3A5, and CYP2D6 and TAM pharmacokinetics were not found.

**Table 3:** Frequencies of CYP2D6 and CYP3A gene variants in the investigated TAM-treated breast cancer patients (*n* = 40)

Gene		Premenopausal (*n* = 20)	Postmenopausal (*n* = 20)
CYP2D6			
Activity score	Function	Frequencies of gene variants	
0	PM	0	0
0.5	IM	0.15	0
1	Slow NM	0.30	0.2
1.5	NM	0.25	0.35
2	NM	0.25	0.35
> 2	UM	0	0
	others	0.05	0.10
CYP3A5			
*3	Variant		
	AA (*3/*3)	0.65	0.7
	AG (*3/*1)	0.35	0.2
	GG (*1/*1)	0	0.1
CYP3A4			
*1b	Variant		
	AA (*1/*1)	0.8	0.7
	AG (*1/*1b)	0.2	0.3
	GG (*1b/*1b)	0	0
*22	Variant		
	CC (*1/*1)	0.9	0.95
	CT (*1/*22)	0.1	0.05
	TT (*22/*22)	0	0

### Multiple regression analysis

Multiple regression modeling was applied to carry out hormonal status-stratified analyses for the premenopausal (*n* = 20) and postmenopausal (n = 20) groups. Initially, we assessed whether the AUCτ and Css mean of TAM and its metabolites differed between the groups based on demographic, phenotype, and genotype variables by univariate analysis. Variables were included in the multiple regression modeling if they had a *p*-value lower than 0.15 in the t-test. The final multiple regression modeling included the hormonal status as the only predictor for Css of TAM [β-coef ± SE, p-value, R^2^ (75.03 ± 17.71, *p* = 0.0001, R^2^ = 0.33)] and 4-HTAM (1.7822 ± 0.4385, *p* = 0.0002, R^2^ = 0.31), while for END, Css included hormonal status (8.578 ± 3.402, *p* = 0.02, R^2^ = 0.32) and race (11.945 ± 2.836, *p* = 0.007, R^2^ = 0.32) as predictors. For the Css of NDTAM, the final multiple regression modeling included hormonal status (286.259 ± 76.766, *p* = 0.0007, R^2^ = 0.37) and weight (− 8.585 ± 3.060, *p* = 0.008, R^2^ = 0.37) as predictors.

## Discussion

The effect of menopausal status on the pharmacokinetics of drugs has been poorly investigated. This is the first study showing the influence of hormonal status on the pharmacokinetics of TAM and its metabolites END, 4-HTAM and NDTAM in pre- and postmenopausal TAM-treated breast cancer patients previously phenotyped as NMs for CYP2D6 and with in vivo CYP3A activity based on midazolam oral clearance [23–25].

In the present study, serial blood samples (*n* = 12) were collected within the dosing interval of 24 h to evaluate the extent of fluctuation in the steady state of TAM and its metabolites. Although the half-lives of TAM and END are prolonged (> 2 days), the data presented in Fig. 1 show that the fluctuation at steady state ranged from 150 to 200% for TAM and its metabolites. Then, the C_ss_ values reported in the present study were calculated as AUCτ/dose interval and not as a plasma concentration collected at only one point within the dosing interval of 24 h. The observed variability in the extent of fluctuation at the steady state of TAM and its metabolites, particularly END, is not only due to genetic factors in CYP enzymes and drug transporter P-gp but also to the simultaneous presence of multiple factors such as race and BMI [5, 31–33].

TAM pharmacokinetics were evaluated in premenopausal patients (*n* = 20) aged 35 to 50 years and in postmenopausal patients (n = 20) aged 60 to 79 years (Table 1). The premenopausal patients had a decrease of 135% in the Css of END and 70–80% in the Css of TAM and its metabolites NDTAM and 4-HTAM (Table 2). These findings are consistent with Lien et al. (1995) [34], who also demonstrated that N-desdimethyltamoxifen exposure was higher in postmenopausal women than premenopausal women, whereas only a tendency was observed for NDTAM.

Based on a multiple regression model, the increased Css of TAM was attributed only to hormonal status, while the increased Css of END was assigned to hormonal status and race (white and non-white), while other demographic characteristics, such as age, were not identified as predictors.

The influence of hormonal status on TAM pharmacokinetics between the investigated groups is probably due to the differences in the bioavailability, considering that the C_SS_ metabolic ratios TAM/NDTAM, TAM/4HTAM, and 4HTAM/END (Table 2) did not change. These results suggest that the increased C_SS_ of the active metabolite END in postmenopausal patients is not due to increased CYP activity but increased TAM bioavailability. Considering that TAM and END are substrates of the efflux drug transporter P-gp [35, 36], it is reasonable to hypothesize that a lower P-gp activity in the gut of postmenopausal patients could result in increased TAM bioavailability.

The influence of hormonal status on TAM pharmacokinetics was not identified in previous studies [37–42], probably due to the lack of balance in the numbers of patients included in both the pre- and postmenopausal groups and the consequent lack of significant statistical power.

Figure 1 shows that the END steady-state plasma concentrations were above 6 ng/mL for 35 of 40 investigated patients. Notably, 6 ng of END/mL plasma is the threshold value associated with a 26% lower risk of recurrence in adjuvant breast cancer treatment [4]. However, when we analyzed the END geometric mean plasma concentrations only in the premenopausal group (*n* = 20), the observed values were too close to the threshold value of 6 ng/mL. This finding is in agreement with Saldores et al. (2015) [15], who demonstrated that patients with low END plasma concentrations (< 6 ng/mL) exhibited a higher risk for distant relapse or death compared with higher END plasma concentrations.

Interestingly, the final multiple regression modeling included not only the hormonal state but also race as predictors for Css of END. In our data, the investigated population was inferred to be white and non-white, with 85% white and 15% non-white premenopausal patients and 75% white and 25% non-white postmenopausal patients. END Css included race as a predictor, with a β-coef of 11.945 ± SE 2.836. The race-associated differences may be a consequence of differences in the frequencies of genetic polymorphisms in drug metabolism and drug transporters, among other factors. This finding is in agreement with Hoffmeyer et al., 2000 [43], who showed that the C3435T SNP of ABCB1 varies among different ethnic groups and correlates with lower expression of intestinal P-gp [44].

**Fig. 3:**
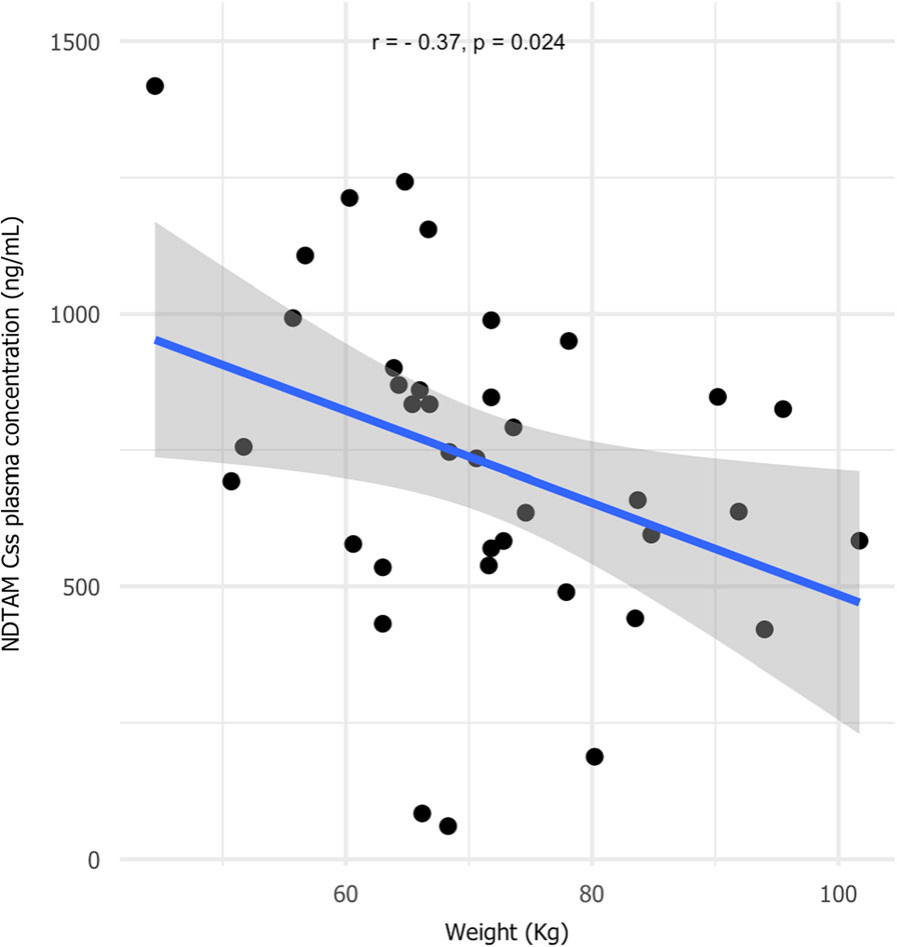
Correlation between body weight and NDTAM steady-state concentrations in all investigated TAM-treated breast cancer patients (*n* = 40)

Previous observations about the relationship between body weight and TAM metabolism are controversial [45]. Regarding the relationship between BMI and plasma exposure of TAM and its metabolites, only the NDTAM Css showed a significant correlation (Fig. 3). This is not surprising since NDTAM is a highly lipophilic agent and has a more prolonged half-life among the TAM metabolites [46, 47].

There were certain limitations to the present study. First, the sample size for the pharmacogenetic analysis was small. Second, a P-gp probe was lacking, and third, limited information from the clinical follow-up was available.

## Conclusions

In conclusion, we identified hormonal status as an essential predictor of the pharmacokinetics of TAM and its metabolites END, 4-HTAM and NDTAM. The premenopausal status was associated with decreased END plasma concentrations by 135% compared to postmenopausal status. Thus, the END plasma concentrations should be monitored mainly in the premenopausal period to maintain plasma levels above the efficacy threshold value.

## Data Availability

On reasonable request, the content is available from the corresponding author.

## Abbreviations

4-HTAM: 4-hydroxytamoxifen
AUC: Area under the plasma concentration-time curve from time zero to 24 h
CL/F: Apparent clearance
Cmax: Maximum plasma concentration
Cmin: Minimum plasma concentration
Css: Concentration in the steady state
END: Endoxifen
ER: Estrogen receptor
IM: Intermediate metabolizer
MR: Metabolic ratio
NDTAM: N-desmethyltamoxifen
NM: Normal metabolizer
PM: Poor metabolizer
TAM: Tamoxifen
Tmax: Time to reach the Cmax
UM: Ultrarapid metabolizer
